# Regulating Professional Conduct by Law

**Published:** 1908-11

**Authors:** 


### EDITORIAL DEPARTMENT.

### REGULATING PROFESSIONAL CONDUCT BY LAW.

**A REMARKABLE DECISION.** **A DISSENTING OPINION.**

Every effort to regulate the practice of medicine has been a failure, in Texas, at least. As soon as one defect in the law is remedied another appears, and it has been found impossible to reach the evil sought—the suppression of quackery. At last, the idea occurred to the Bureaucrats at Chicago, who run the machine, to join forces with all the irregulars and make common cause against the hated Christian Science people and suppress *them,* at least. Hence, a pattern bill was cut out and sent to the State Associations, and the mixed board bill is the result—fish, flesh, fowl and good red herring—and several States have bills cut by that pattern. Oklahoma's is almost identical with that of Texas, so much so that the comments of the editor of the Oklahoma *Medical News-Journal* on the law fits the case in Texas—as far as it goes—and I reproduce it *verbatim.* So, while the entire push, regular, irregular and defective, sat down with a heavy dull thud on the Christian Science people, they left the door open to Dr. Caius and his sort, and the voice of the quack, like that of the turtle in scripture, is heard in the land, loud and long.

The comic supplement in the papers every Sunday picture a Swede woman trying to kill a cat. In every instance the cat slips by her, and she stumbles over it. We are very much in the position of "Yenevieve Yonson," the Swede woman, and the quack is the cat. We whack away at Dr. Caius, whose advertisement in the papers of the Capital of Texas, plainly show flagrant, gross unprofessional conduct, and fraud, deception and false statements, but he dodges the lick, and trips us up. When the Board, acting under the plain letter of the law, requested the county or district attorney to bring suit to revoke the license of "Dr. Lafayette," the "phenomenal," notwithstanding it is mandatory and not discretionary, the attorney failed or refused to do so. "There is a reason," as the coffee substitute advertisers say. Popular sympathy is with the quack. The people *will be* fools and suckers; they *will be* dupes,—and they are,—and it would make the attorney unpopular (looking ahead to re-election) to prosecute the quack who has amused and incidentally fleeced the Austin people all summer. It was up to the Board, therefore, to resort to mandamus to make them or one of them do his duty. (Read Section 12, appended.)

But, before instituting such proceeding, a member of the Board thought he should make sure of his ground, and (the Board having no money) find out what such a suit was going to cost. Hence he asked the Attorney General, who, in case of failure of the attorney to convict, was to pay the cost, and, *mirabile dictu!* Assistant Attorney General Sluder says the Board must pay the cost, if they have the money, and, if they have not, the courts must wait until they get it. But he does not say what will be the result if they never get it; nevertheless, the Board may be sued for it, he says.

I reproduce below this remarkable opinion from the *Texas State Journal of Medicine* for October.

It is the most remarkable decision on record, with perhaps one single exception, in the annals of jurisprudence, and it is like unto it, except that it is a "little more so." Its prototype and precedent (referred to) is found in Washington Irving's Knickerbockers' History of New York. It was the memorable case of Wandle Schoonhoven vs. Barent Blecker. Blecker owed Wandle a heavy balance and refused to settle. The old Dutch Burgher, Wooter Van Twiller, was Governor of the Nieuw Nederlandts, the capital being New Amsterdam, now New York. Irving says:

"Having listened attentively to the statement of Wandle Schoonhoven, giving an occasional grunt, as he shoveled a spoonful of Indian pudding into his mouth, either as a sign that he relished the dish, or comprehended the story, he called unto him his constable, and, pulling out of his breeches pocket a huge jack-knife, dispatched it after the defendant as a summons, accompanied by his tobacco box as a warrant. * * * The two parties being confronted before him, each produced a book of accounts written in a language and character that would have puzzled any but a High Dutch commentator or a learned decipherer of Egyptian obelisks to understand. The sage Wooter took them one after the other, and having poised them in his hands and attentively counted over the leaves, fell straightway into a very great doubt, and smoked for half an hour without saying a word. At length, laying his finger beside his nose and shutting his eyes for a moment, with the air of a man who has just caught a subtle idea by the tail, he slowly took his pipe from his mouth, puffed forth a column of tobacco smoke, and with marvelous gravity and solemnity pronounced that, having carefully counted over the leaves and weighed the books, it was found that one was just as thick and as heavy as the other; therefore, it was the final opinion of the court that the accounts were equally balanced; therefore, Wandle should give Barent a receipt, and Barent should give Wandle a receipt, and the *constable should pay the costs!*"

Seriously, if this decision "goes" as the proper interpretation of the law (see Section 12, herewith), it effectually blocks the game—protects the quack and puts the Board to shame. They are powerless. They have no money. It defeats the purpose of the law. No provision is made in the law for prosecution of quacks *by the Board;* it is not the Board's business to prosecute the case but only to request the county or district attorney to bring suit. If the suit is to revoke a license for gross unprofessional conduct, advertising that is fraudulent and calculated to deceive or for other reason than violating the law (which would necessitate criminal proceedings), it is a civil suit and a *State case;* the State is the plaintiff and the accused is the defendant; the Board is only the informant; and while the complaining member must appear as a witness, he is in no way—nor is the Board—responsible for the costs, any more than I would be, or any other citizen, who puts the authorities "next," say, reports the infraction of any statute, civil or criminal. If such opinion is to hold, no one would dare inform against any criminal, or offender against any civil statute. He would be in the position of the poor constable—and worse; he may be sued for costs.

It may be thought presumptuous in me to thus dissent from the highest authority in the State, but it seems to me to be so plain a case that the expense of prosecuting a quack under the provisions of the law (should the State fail) should be borne by the State that I must think that Judge Sluder has not been properly quoted.

It seems that no two judges construe the law alike. In Tarrant application for mandamus against the Board was granted, and in McLennan it was refused; in Harris, granted; in El Paso, refused, and so it goes.

Let us repeal the law and get a better one—or none. It is a failure so far as suppressing quackery goes. The cat takes a fall out of us every time.

Here is Section 12 of the One Board Law to regulate the practice:

"The right herein to practice medicine in this State may be revoked by any court of competent jurisdiction, upon proof of the violation of the law in any respect in regard thereto, *or for any cause for which the State Board of Medical Examiners is au-*

*thorized to refuse to admit persons to its examinations* as provided in Section 11 of this act; and it *shall be the duty* of the several district and county attorneys of this State to file and prosecute appropriate judicial proceedings *in the name of the State* (italics ours) on request of any member of said Board."

Dr. Foscue of the Board requested the district attorney of Travis county to bring suit to revoke the license of one Dr. Berry (the "Dr. Caius" in this instance)—calling himself "Dr. Lafayette" and "The Phenomenal"—*not* for violation of the law (for he has a license), but for "grossly unprofessional * * * conduct of a character likely to deceive or defraud the public," as provided in Section 11—one of the causes for which the Board may refuse a license. The district attorney, like a fool, took it to the grand jury who, very properly threw it out—it not being an indictable offense, but added that it was "an effort to down a great man."

We do not know whether or not Judge Sluder "straightway fell into a very great doubt," but here is his decision as to the costs. It will be noted that he hints at suit for the collection of the costs. This will estop any attempt by the Board to suppress quackery—to revoke a license for unprofessional conduct, and here's where the constable had the better of the Board. Rip Wooter Van Twiller didn't sue him—so far as heard from.

From *Texas State Journal of Medicine,* October, 1908, page 160:

"I am of the opinion that in litigation involving the construction, the enforcement or the validity of this medical act, in cases where the Board loses in such litigation, that the expense of such litigation would have to be borne by the Board, provided the Board had funds in its hands belonging to the Board officially, sufficient to satisfy such cost. Unless the Board had such funds or should thereafter collect funds sufficient to satisfy such costs, it occurs to me there would be no way of satisfying the cost accruing from such litigation. The members of the Medical Board would be acting in their official capacity, and they would be sued as officials and not as individuals, and no judgment could be properly rendered against them individually so as to subject their individual property to the payment of any costs of litigation, or any judgment that might be procured against such Board. But, of course, as stated above, all funds coming into their hands as official funds authorized by law to be used by the Board in paying expenses of the Board could properly and legally be appropriated to the payment of such costs.

Yours very truly,

J. T. SLUDER,

Office Assistant Attorney General.

Austin, Texas, August 12, 1908."

The Board need not employ a lawyer: Dr. Chase (*loc. cit.*) calls on the profession to ante to create a fund for the Board to pay a lawyer. Judge Sluder says (*loc. cit.*):

"This Department has decided to furnish a representative to the Medical Board in all cases involving the construction and the validity of that medical act."

Here's a pretty howde do. And here's Dr. Phelan's construction of the Oklahoma law, showing that the Oklahoma law and the Texas law are cut by the same pattern, without doubt—sent from Chicago headquarters:

"Revoking Licenses for Advertising: So many physicians are asking concerning the status of advertising under the recent resolutions of the Examining Board that the following statement may be of interest. The State Medical Examining Board under the new law can not revoke a license for any cause. The right to practice may be revoked only by a court. It is the duty of district and county attorneys to file suit and prosecute the same, in the name of the State, on the request of any member of the Board. In order to have a license revoked, it must be shown that the defendant is guilty of some of the acts for which the Board is authorized to refuse to admit persons to its examinations, namely, fraud or deception in obtaining licenses, conviction of a crime of the grade of felony or one which involves moral turpitude, or procuring, or aiding or abetting the procuring of a criminal abortion, or other grossly unprofessional or dishonorable conduct of a character likely to deceive or defraud the public, or for habits of intemperance or drug addiction calculated to injure the lives of patients. The Board has no such right, and will only have suit entered where advertisements are of a character likely to 'deceive or defraud the public,' such as the guaranty of cures or the claim of unusual power for the cure of incurable diseases. The promising or advertising of 'No cure, no pay,' is unprofessional, bad business, and bad common sense, but it does not furnish ground for legal action for revocation of license."—*Oklahoma Medical News-Journal.*

It will be remembered that the "Dr. Caius" who has been in Austin all summer with a vaudeville troup, singing, dancing and curing appendicitis while you wait, removing *hats* full of gall-stones, killing cancers, etc., between acts, is under indictment by the Federal grand jury at Houston for fraudulent use of the mails, and is under $1500 bond to appear for trial by Federal court in February. It is thought that he took $50,000 out of Travis county. Alas, "our noble profession."

N. B.—Wanted—An opinion on the status of Osteopaths under this law. They had no legal status under any previous law, being exempt from all law because they "give no medicine."

